# Awareness of parental illness: a grounded theory of upholding family equilibrium in parents on long-term sick-leave in primary health care

**DOI:** 10.1080/02813432.2021.1928835

**Published:** 2021-06-21

**Authors:** Charlotte Oja, Tobias Edbom, Anna Nager, Jörgen Månsson, Solvig Ekblad

**Affiliations:** a Department of Neurobiology, Care Sciences and Society (NVS), Division of Family Medicine and Primary Care, Karolinska Institutet, Stockholm, Sweden; b Department of Clinical Neuroscience (CNS), Center for Psychiatric Research, Karolinska University Hospital, Stockholm, Sweden; c Department of Public Health and Community Medicine/Primary Health Care, Institute of Medicine, Sahlgrenska Academy, University of Gothenburg, Gothenburg, Sweden; d Academic Primary Health Care Center, Region Stockholm, SLSO, Stockholm, Sweden; e Department of Learning, Informatics, Management and Ethics (LIME), Cultural Medicine, Karolinska Institutet, Stockholm, Sweden

**Keywords:** Child of impaired parents, primary health care, sick leave, parent–child relations, family health, qualitative research, health promotion

## Abstract

**Objective:**

To understand the main concern of chronically ill parents and how they resolve this concern in relation to their children.

**Design:**

Grounded theory.

**Setting:**

Three primary health care clinics in Sweden.

**Subjects:**

Thirty-two interviewed parents and their children.

**Main outcome measures:**

Processes and typologies of upholding family relationships.

**Results:**

A concern of chronically ill parents is *sustaining family equilibrium*, achieved through a process of *upholding family relationships*. How a parent upholds depends upon his/her *comprehension* of the illness and of their child’s *need for parenting*. In response to the parent’s upholding behaviours, children *mirror* the effect of the illness to the parent, the child’s specific behaviour depending on his/her level of comprehension regarding the parent’s illness. Their combined behaviours create an *awareness context* that may be *closed, concealed, suspicious, conflicted, mutual pretence* or *open*.

When the parent drives and facilitates the evolution of comprehension, the context quickly evolves from closed to open. When the parent hinders the process by *masking* and *resisting* the child responds by *probing* and *proving* and they become locked into a suspicious or conflicted awareness context with high relational tension. To create family equilibrium the parent needs to reveal and facilitate the awareness process.

**Conclusion:**

Parents on long-term sick leave in primary health care can need assistance to facilitate the awareness context of themselves and their child.

**Implications:** Clinicians can identify the current awareness context of their patient and help their patient towards increased understanding of their illness; their child’s needs and the parental capacities needed to reveal the illness and its impacts.

**Key Points**
- Children are affected when parents are ill; they wish for information on their parent’s illness. Effective interventions are available in settings other than primary health care and possibilities seen by GPs and families in Scandinavian primary health care have been previously described. There is a knowledge gap in how parents view themselves and their parenting when ill in primary health care. An analysis grounded in interviews was needed to generate a hypothesis (theory) of parental concerns and behaviours.
- This theory proposes that an important concern of chronically ill parents is to sustain family equilibrium, which they attempt to do by upholding family relationships.
- Specific upholding behaviours include masking, resisting, colluding, and revealing. In response, children will engage in mirroring behaviours. Which paired behaviours are enacted will depend upon the respective levels of comprehension of parent and child regarding the illness and on the child’s need for parenting. In their interactions, parent and child create one of six awareness contexts.
- Identifying the current awareness context in the family about chronic parental illness provides clinicians with a conceptual tool to better support those families locked in suspicious or conflicted awareness contexts.

## Introduction

Children are often aware when parents are ill. This has been shown in primary health care where children with chronically ill parents view their own situation as very difficult. The children wish their parents could answer their questions and help them understand the parental illness [1]. To understand the parental side of this situation we will explore the main concern and strategies of these children’s parents in relation to their own children.

There is no published calculation of how many children in Sweden are affected by parental chronic illness in a primary health care setting. A report describing children of parents hospitalized for at least a week [2] concluded that this affects 25.7% of children in Sweden (12.6% somatic illness, 5.7% psychiatric illness, 3.4% death of parent, 2.5% alcohol dependence and 1.5% dependence of narcotics). Many more children can be assumed to have parents with illnesses not demanding hospitalization for a week or more. Recognition that children in families affected by parental illness have a heightened risk for illness [3] and other poor life outcomes has resulted in the creation of psycho-educational interventions [4–8] to reduce the negative effects of the parental illness on the children and to support children’s healthy development. Our systematic review of such interventions in all health care settings globally [9] concludes that research so far has been conducted in mental health settings, including substance abuse, and in cancer and HIV care. The studies showed a small-to-moderately-positive statistically significant intervention effect on the children’s level of internalized and externalized symptoms. One of the interventions, the Beardslee family intervention, was shown to be effective in Scandinavian psychiatry [10] and well accepted by families [11]. Content analysis in our review [9] resulted in four concepts important to both children and parents in the interventions; increased knowledge, more open communication, new coping strategies and changed feelings. Three additional concepts important to parents have observed changes in their children’s behaviour, parents’ increased understanding of their own children and the relief of respite.

From a perspective of law and policy, there are also impelling reasons to focus on how to better protect the health of growing children. The third of the global goals of the UN’s 2030 Agenda for Sustainable Development challenge all to ‘ensure healthy lives and promote well-being for all at all ages’ [12]. The United Nations Convention on the Rights of the Child [13], which became law in Sweden on 1 January 2020, states that children should enjoy rights including the right to be listened to, and should be given special protection, opportunities and facilities. In addition, some nations have national regulations guiding health care staff with adult patients to attend to the needs of these patients’ children. For example, in Sweden [14] healthcare personnel are required by law to give information, advice and support to under-aged children whose parents are seriously mentally or physically ill, have an addictive disorder or have died. Similar legislation exists in Denmark [15], Finland [16] and Norway [17].

Primary health care is a specific discipline with a need for specific research because it has unique epidemiology, the context of care is important, and it has a strong link and responsibility to the community [18]. According to the Health and Medical Care Act [19] primary care specifically provides health and medical care services for both common physical and mental health care needs. In 2020 there were 159,000 ongoing sick leave cases in Sweden, 103,000 women and 56,000 men. Benefits were given due to mostly psychiatric diagnosis (51%) (including stress 22%), injuries (13%) and musculoskeletal illness (10%) [20]. Many of these patients are cared for in primary health care where more than half of all visits in Swedish health care are done [21]. The primary health care focus on prevention (of illness in the children), detection and treatment of the ill parent (and perhaps the children) and family and everyday life, may provide an opportune arena for assisting to mitigate the effect of parental illness on children.

There is some scientifically grounded knowledge in Scandinavian primary health care from the perspective of professionals, parents, and their children.

Norwegian general practitioners (GPs) saw that they could help children of patients by identifying children at risk, counselling the parents, and collaborating with other healthcare professionals and social workers. But there were several barriers to doing this: time constraints, the GPs main focus on only the patient present in a consultation, and GPs being afraid of hurting or losing their vulnerable patients thus avoiding bringing up the patients’ children as a subject for discussion [22]. GPs’ perceived mandate of trust from the parents was a precondition for the children’s situation to be addressed. Some GPs took an open mandate from the parent for granted, while others assumed that the parent did not want to discuss their family situation [23].

Adolescents with ill or substance-abusing parents in primary health care interviewed in Norway and Sweden experience unpredictability in life and strive to find a balance between their own needs and the restrictions caused by parental illness and they sought a GP most often for somatic complaints [24]. They struggled to make life work for both themselves and their parents by trying to understand the situation and by adopting the parental role [1].

Parents provide childhood experiences and populate the environments that guide children’s development and so contribute to child mental health. Parenting is expressed in cognitions and practices [25]. Ill parents in primary health care in Norway were found to convey a double message to their helpers. They wanted to be considered responsible and well-intentioned parents who wished the best for their children. At the same time, they needed support in parenting [26].

Lacking is scientific knowledge exploring the complexity of what happens between parents and their children in a primary health care setting. This is important when developing better care for patients.

## Objective

The objective of the present study was therefore to explore the main concern of parents on long-term sick leave in primary health care and how they resolve their main concern in relation to their children.

## Design

The mainly qualitative research method, grounded theory, developed by Glaser and Strauss [15–18] was used to generate conceptual understanding (theory) from a bottom-up analysis of interviews in a previously under-researched field. The theory is not merely a description of the research field but may be used to predict likely future behaviour. The research team worked from a constructivist/interpretivist research paradigm, acknowledging the inevitable impact the researcher has on the interpretation and analysis of the data.

We all have preconceptions and can, according to Charmaz, reveal them to ourselves by being intimately familiar with the phenomenon we study [27]. All the authors are intimately familiar with the context and phenomenon under study. As a GP at the same clinic for 18 years the first author is familiar with the population and the clinic. As a private person, she also has experience of motherhood and illness. The last author works in primary health care and was the main supervisor of the students who performed the interviews, providing theoretical sensitivity in the analytic phase.

## Material and methods

Interviews with children and their parents explored the experiences of children as next of kin in primary healthcare. An article analysing the perspective and experience of children (11–16 years old) of chronically ill parents in primary health care is reported elsewhere [1]. This data is re-analysed from the perspective of parents for the present article together with data from the second wave of interviews with parents and children.

### Context, sample, and interviews

Primary health care in Sweden is the first contact for all non-emergency cases. A very broad set of patients and health problems are handled, including being the first level of psychiatric care. Patients with chronic illness are often also investigated in secondary care and if no specific treatable disease is found, returned to the primary health care clinic for continued management. Patients treated in secondary care would be sick-listed from those clinics and not from the primary health care clinic and would not be found and invited to this study.

Seriously ill patients in primary health care have diverse and multiple illnesses often including a combination of psychiatric and somatic symptoms as well as social and economic risk factors. The severity of the illness is usually not defined by the diagnosis or mortality, but rather by a lack of everyday function over an extended period. Sick leave, rather than diagnosis, was therefore chosen as the inclusion criterion of the study. Patients on sick leave, defined as certified medical inability to work for more than 90 consecutive days, thereby excluding parents with minor and quickly-healing illness less likely to influence the children. To assure that they were parents of children under the age of eighteen the children of these patients were identified *via* the Swedish Tax Agency. Parents were invited to the study by their doctor. Children were invited to the study by their parents. Parents and children came together to the clinic, were informed together, and had the opportunity to ask questions before parents and children signed forms of consent. Interviews were conducted with parents and children independently to reduce the influence of the presence of the other one. As one was interviewed the other waited in an adjacent room with fruit and colour pens to pass the time. After the interviews, all were asked if they had any further questions.

#### Sample in the first wave of interviews:

Initially, fourteen parents (11 mothers and 3 fathers) were interviewed at a primary health care centre in Stockholm, Sweden.

Semi-structured interviews were conducted individually in a GP surgery at the patient’s primary health care centre PHC during February and March 2015. Total interview time was almost 19 h, with a mean time per interview of 78 min. The interviews were conducted by two medical students writing their master’s theses [28,29] and semi-structured, loosely following an interview guide (Appendix 1). In a calm room at the clinic, participants were invited to share their experiences and discuss how parenting can be introduced in a dialogue between parents and staff.

#### Sample in the second wave of interviews:

The second wave of interviews was conducted at the first primary health care clinics and in clinics in two additional suburbs of Stockholm. In this wave of invitations, 18 of 53 initially invited parents consented and took the time to be interviewed. Those who declined often gave a lack of energy and time as the reason. The children of these 18 parents were invited *via* their parents and 15 children consented and were interviewed. The first clinic was again in the suburb south of Stockholm city centre with a slightly, for the city of Stockholm, lower than average socioeconomic status (Care Need Index CNI 1.3) [30]. In the first clinic psychology students doing their master’s thesis [31] conducted interviews number 1 to 8 in February and March 2019, to avoid the first authors' personal acquaintance with the patients influencing the answers. The second clinic was in a northern suburb with one of the lowest socioeconomic statuses in Stockholm (CNI 1.9). In the second and third clinic interviews were conducted by GP’s in residency training [32,33]. The third clinic was located just south of the city centre and has a high socioeconomic status (CNI 0.7). The median age of the parents in the second wave of interviews was 46 years with a range of 28 to 56 years. Further demographic data of the second wave of interviews can be reviewed in Table 1.

**Table 1.** Demographic data of study participants in the second wave of interviews.

				Child age
Interview (number)	Clinic	Parental gender (mother/father)	Child gender (son/daughter)	Average 12 (7–17) (years)
1	1	Mother	Son	12
2	1	Mother	Daughter	13
3	1	Father	Son	16
4	1	Mother	Not interviewed	
5	1	Mother	Daughter	11
6	1	Father	Not interviewed	
7	1	Mother	Daughter	16
8	1	Mother	Not interviewed	
9	2	Mother	Son	17
10	2	Mother	Daughter	10
11	2	Mother	Son	16
12	2	Mother	Son	15
13	2	Mother	Daughter	11
14	3	Mother	Son	8
15	3	Father	Son	11
16	3	Mother	Son	7
17	3	Mother	Son	9
18	3	Mother	Son	10

Again, the interviews were semi-structured, loosely following an interview guide (Appendix 2) enabling them to freely share their wishes for when, where and how children could be informed of their parents’ illness and what support they would like from the clinic. Total interview time for parents and children was now 20.5 h, with a mean time per interview of 68 min. All the interviews were conducted in Swedish, audio recorded, immediately anonymized, and transcribed into Word documents.

### Analysis

The transcribed interviews were imported by the first author into NVivo 11 and open inductive coding was conducted manually. Using the constant comparison method, [27] concepts were gradually emerged. Memos were written continuously. Focused coding explored key codes such as Striving to act as before, Feelings of the parent, Own childhood, Interaction between parent and child, Transformation because of illness, Verbalizing/speaking to child and more. The codes were then related to each other until the main concern of struggling to handle parenting while ill emerged with important codes such as confused parental understanding and weakened parental role emerged [34–36]. To achieve theoretical completeness, it was found that theoretical sampling was required and that more data exploring the characteristics and time processes of the struggle of parenting when ill, were necessary. The second wave of interviews was therefore conducted and the data analysed. New concepts emerged such as parental silence and inactivity and child activity and supportiveness, masking symptoms and child needs and child noticing and accepting before the parent. At this stage methodological mentoring was sought at Grounded Theory Online to raise the level of conceptualization and structure the theory. As the codes at this point were well defined and no new codes emerged saturation had been reached. In the process of theoretical coding, there was found to be an emergent fit with Glaser’s core category of awareness contexts, previously discussed in Awareness of Dying [37]. In a process of constant comparison and sorting of memos, the theory was refined and now conceptually explains what is going on in the area of study.

### Research ethics

Studying parents and their children in primary health care is ethically challenging. Benefits include generating new and critically needed knowledge, while ethical risks include putting negative stress or stigma on already vulnerable patients and their children [38]. After considering the ethical dimensions, this study was deemed important enough to conduct and was approved by the Stockholm Regional Ethical Review Board (2013/62–31/5, 2014/1454–32/5 and PUL KI 2–3750/2014). The parents and children were all informed of the research in writing and orally and had the opportunity to ask questions before signing forms of consent. Data security was upheld.

## Results

The main concern of chronically ill parents is sustaining family equilibrium, which they attempt through a process of upholding family relationships.

‘I struggle every day … I fool myself, fool my family, fool all, trying to stand there and show that I can do it’. Parent 9.

The illness disrupts the life of the parent absorbing much of their time and energy. As the symptoms of illness and the decreasing capacity of the parent impact family life, the parent may not be aware that his/her child is noticing these changes. Neither does the parent notice the child’s need to comprehend what is happening. Instead, the parent focuses on upholding previous parenting and communication behaviours in an attempt to normalize the situation and sustain family equilibrium.

In the face of upholding behaviours and unrecognised needs, the child’s need for active parenting motivates the child to act: the child begins to mirror the illness to the parent in an attempt to engage the parent in meaningful discussion. Taken up with their own concerns, however, some parents are slow to recognize that their upholding behaviours are inappropriate in a deteriorating situation. Subsequent interactions between parent and child can cause relational tensions, which bring the family into disequilibrium and potentially chaos. The interactions of parent and child will also create one of six contexts of awareness of illness: closed, concealed, suspicious, conflicted, mutual pretence or open (Table 2). Understanding the current awareness context of the family provides a conceptual tool that can be used to support parents locked in non-optimal contexts and enable them to parent their child in the way that that the child yearns for.

**Table 2.** Illness induced relational change: awareness contexts of parent and child.

Awareness context	Closed	Concealed	Suspicious	Conflicted	Mutual pretence	Open
Child’s comprehension of parental illness	Low	Low	High	High	High	High
Parent’s comprehension of illness and child needs	Low illness Low child need	High illness Low child need	Low illness Low child need	High illness High child need	High illness High child need	High illness High child need
Parent’s behaviour: *Upholding family relationships*	**Upholding** Not noticing illness Not noticing child need	**Masking** Accepting illness Denying child need	**Upholding** Unconvinced of illness Denying child need	**Resisting** Accepting illness Accepting child need	**Colluding** Pretending illness Pretending child need	**Revealing** Understanding illness Understanding child need
Child’s behaviour: *Mirroring*	**Noticing illness**	**Noticing illness**	**Challenging**	**Challenging**	**Colluding**	**Contributing**
Relational tension	Low	Low	High	Very high	Medium	Low
Family equilibrium	Equilibrium	Equilibrium	Disequilibrium (slight)	Disequilibrium (severe)	Equilibrium (locked up)	Equilibrium (dynamic)

### Awareness contexts

Table 2 shows that the awareness context is a function of the level of comprehension regarding the parent’s illness of both parent and child; the parent’s level of comprehension of the child’s need for facilitative parenting; and the respective upholding and mirroring behaviours of the parent and child.

#### Closed and concealed awareness contexts

In both the closed and concealed awareness contexts, the child begins to notice changes in the parent: noticing symptoms and loss of function. The child also notices that family life and social life are in some way compromised, perhaps noticing that their home is less well cared for. The child, however, is unaware that these impacts are caused by illness.

In a closed awareness context, the parent is also unaware of the illness and is upholding previous behaviour as if the illness or the child’s needs have no impact. In a concealed awareness context, the parent has a level of comprehension regarding his/her illness and seeks to masks or conceal its effects, verbally rejecting their child’s noticing, probing and proving statements, insisting that things are fine or not so bad. The relational tension is low, and the family relations are in threatened equilibrium.

#### Suspicious and conflicted awareness contexts

In both the suspicious and conflicted awareness contexts, the child begins to understand that the parent is ill. In a suspicious awareness context, however, the parent remains unconvinced both of his/her illness and of the child’s needs in relation to the illness. The parent continues to uphold behaviours and deny the child’s needs. In response, the suspicious child challenges the parent, by probing for more information: verbally stating parental symptoms and asking questions in the hope that the parent will reveal the nature of the illness and the shape of things to come. Since the parent does not reveal, the child also seeks to prove the parent’s diminishing capacity and family disequilibrium: meaning that the child tries to contribute to the awareness process, by pointing out facts and explaining and contextualizing the parent’s symptoms to the parent.

In a conflicted awareness context, the parent understands both the nature of his/her illness and the child’s needs but uncertain of their own competence to engage with the child, resists the child’s challenges: actively denying or diminishing their child’s changing awareness and their own symptoms and loss of function. The relational tension is high or very high and the family relations are in slight or severe disequilibrium.

‘My daughter says: Mom, why are you sad again? I say: No dear, no, I am not sad’. Parent 12

#### Mutual pretence contexts

In a context of mutual pretence both the child and parent are aware of the illness and the child´s needs. The parent invites the child to collude by pretending either that they do not know, or that what they both know does not matter. The child accepts the invitation and colludes to avoid further erosion of parental role or declines to collude and they remain in the conflicted awareness context. If the child colludes the relational tension decreases somewhat, and the family is in a locked-up equilibrium.

#### Open awareness context

In a context of open awareness, both parent and child understand the nature of the illness and the child’s needs. The parent engages in discussion with the child to reveal and acknowledge the illness. Under these circumstances, the child can cease any previous challenging or colluding behaviours and contribute to family relationships such that relational tension is low, and the family is in dynamic equilibrium.

### How the parent–child awareness moves from one context to another

All families will experience the closed awareness context and at least one other: which other contexts are experienced varies.

The way in which the awareness contexts evolve from one to another mainly depends on: (i) the parent’s characteristics (ii) the pace of development of the parent’s comprehension of the parent’s illness by the parent and; (iii) by the child and: (iv) on the pace of the parent’s changing understanding of the child’s need for parenting. Two types of parents emerged: ‘Facilitators’ and ‘Resisters’.

#### Types of parents

Once the illness is noticed both parent and child wish the parent to oversee the process of increasing awareness with the child, in particular, yearning for the stability that active parenting brings. Parents who manage this process are Facilitators.

Facilitators are characterized by having higher degrees of self-worth; self-knowledge; comprehension about their illness; parenting skills; emotional stability; and have a higher need and hence motivation, to facilitate their child’s comprehension. The greater the degree to which they possess these characteristics the better they are likely to be at facilitating the awareness of their child. Importantly, having the words to explain, the patience to endure and the emotional capacity to support the child’s need for the parent to remain a parent, differentiates the Facilitator from the Resister.

The Resister is characterized by having lower degrees of self-worth; self-knowledge; comprehension about their illness; parenting skills and emotional stability. These parents also need to support their child but recognise that they lack the capacity and ability to do so in the way the child needs: their pain is compounded.

‘To wish to be there for once child, but not be able to, is the most difficult of all’ Parent 4.

#### Ideal process though awareness contexts: driven and facilitated by parental awareness

An unproblematic pattern of evolution of awareness is that of moving from closed to open awareness. Here the relational tension remains low and family equilibrium is maintained. This ideal process clinically happens but is rarely noted by the clinician, as there is no prolonged problem situation to observe. The facilitating parent comprehends and accepts the illness and child's needs. The child has an age-appropriate need for awareness development and therefore has a natural drive to understand themselves, their parent, and the relationship. The parent propels the awareness development by soon revealing a reality-based understanding, of the illness and the child’s needs, to the child and they enter an open awareness context together. The parent is motivated to reveal it as they consider it a part of their parenting responsibility.

‘Interviewer: So, what makes you go on? My stubbornness and my children’. Parent 14.

The parent has the capacity to welcome what the child notices and to answer their questions. The child remains relationally calm and contributes in the context of open awareness.

#### Variation from the ideal process: driven by child awareness and the parent resist the awareness process

Driven by the child’s increasing comprehension, more problematic patterns of evolution of awareness are from closed or concealed awareness contexts to suspicious or conflicted contexts.

In these contexts, relational tension grows high and the family is in disequilibrium, sometimes severely so. It is a common clinical situation, noticed by clinicians, as there is a prolonged period of tension to observe.

The Resister does not initially comprehend nor accept the illness nor the child’s needs. The child is noticing and is trying to move the awareness context by probing and proving. The parent doubts his/her capacity to reveal and so hinders the development of awareness by masking or resisting. The child strives for increased awareness as part of a general, age-appropriate need to know. The more parent resists and the child challenges, the more they become locked into a conflicted awareness context. As the parent does not have the capacity to welcome what the child notices nor answer their questions, the child becomes relationally upset and the high relational tension feeds into the awareness context. The relational tension is very high, and the family is in disequilibrium.

‘The greatest difficulty is not accepting one’s situation. Denial, denial’. Parent 3.

Both child and parent are trying to sustain the family equilibrium and uphold family relationships in a context of increasing awareness of illness and child needs, but by using different strategies. In mirroring, the child is trying to sustain the family equilibrium by noticing, proving, and probing to move the common awareness context to open.

The parent is using two different behaviours to sustain family equilibrium: masking and resisting. Parents and children can remain in a suspicious or conflicted awareness context for a prolonged time (months, years, and decades). They do, however, have the option of colluding to create a context of mutual pretence or the parent has the option of revealing and moving them both into an open awareness context.

### Context of mutual pretence

To reduce relational tension and increase family equilibrium the parent and child might choose to collude, to pretend that they do not know, or that what they know does not matter and to uphold a mutual pretence that all is well. The parent invites the child to collude and the child might agree so as not to lose a positive parental figure psychologically and socially. If the child declines to collude, they will remain in the conflicted awareness context. A context of mutual pretence can be maintained for a prolonged period (months, years, and decades).

#### How to escape suspicious, conflicted, or mutual pretence awareness contexts: by revealing

To create a dynamic family equilibrium the parent needs to reveal and become a facilitator of the awareness process. To do so, these parents need high degrees of self-worth.

‘I need your support, from both doctors and psychologists. I wish you to remember that’. Parent 3.

The child wishes their parent to reveal the symptoms and diagnosis, the treatment, the prognosis, the inner feelings of the parent and the self-understood life story of the parent.

‘The sick one should tell, the one with the secret’. Child 7.

Ideally, revealing should take place in a safe, relaxed, and calm atmosphere, preferably privately in the family home or together with professionals at home or the clinic. There are different techniques for revealing such as passively nodding when the child states something, verbally confirming what the child states and mandating others to reveal on the parents’ behalf. The children wish their parents to reveal actively to them by telling them what is going on, inviting them to converse and answer questions. If this is not possible for the parent, the children accept passive revealing.

## Discussion

Patients sometimes seek primary health care when the family is in a state of disequilibrium and even chaos. In that clinical situation, it is important for diagnosis and treatment to understand the important influences in the patient’s life causing tension and disequilibrium. This study reveals a potential and important contributing factor to tension and disequilibrium in patients who are parents of underaged children. For professionals, this theory highlights that by helping the parent to a higher degree of comprehension we also assist the child. Our findings are congruent with those of primary health care in Norway [26] where parents were found to convey a double message to their helpers, in wishing to be considered responsible and well-intended parents and at the same time needing support in parenting. Our findings contribute a theory explaining what underlies the need to convey such a double message.

To be a child of an impaired parent is a recognized risk factor for the health of the child. This study clarifies that also in primary health care there are patients whose children are strongly impacted by their parent’s illness.

‘I think this is something serious people should know more about’. Child 9.

Teenagers have an age-appropriate drive for increased awareness and if it is not possible for the parent to welcome their child’s growing awareness the parent might unnecessarily interpret the child’s behaviour in too negative a light. For many children who grew up or are currently living in families with chronically ill parents, this theory may assist them to understand their experience. Recognition that they are not alone in their experience and were not especially difficult or confused as teenagers, might contribute to self-understanding and their feeling of self-worth. These feelings are valuable assets as they may soon become parents themselves.

Upholding family relationships, in a context of increasing awareness of the parental illness and child needs, emerged as an explanation as to what parents do to sustain family equilibrium. Awareness contexts are, according to grounded theory taxonomy, a theoretical code contextualizing how the substantive codes related to each other [34]. Movement in different types of awareness contexts is an important basic social process and emerged as the theoretical code in Glaser and Strauss's important work “Awareness of dying” [37]. Awareness contexts are a conceptual tool independent of the subject under study. It has been used previously to explore awareness of dementia [39–41]. Awareness contexts are also likely to be helpful when thinking of interactions between parents and children in different types of parental difficulties, such as economic vulnerability and divorce.

### Limitations

This study explains the interaction of one parent and one school-age child. In further studies, it would be helpful to explore the behaviours and interactions of the parent of babies and pre-school children. Also, many families have more than one child with a smaller or larger age difference between the children and this is likely to change the behaviours and relationships. It would also be important to understand the presence or absence of a healthy parent as well as the influence of the gender of the parents and children.

### Contribution to policy and practice

This conceptual theory is useful for clinicians and researchers. It can be used to understand in which awareness context a patient currently is operating. This understanding enables professionals to hypothesise the position of that parent’s child. We can explore what capacities the parent already possesses and what capacities may need to be supported to enable the parent to reveal to their child. As support to develop these capacities is often unavailable, parents are unable to reveal and the parent and child may stay locked in suspicious, conflicted, or colluding awareness contexts, keeping the family in disequilibrium and demanding continuous input of energy from both the parent and child. The health of both could benefit from this energy being spent on developing healthy family relationships instead. Further research is needed on the suitable support clinicians can render ill parents to enable them to become Revealers. Thereafter resources suitable for primary health care need to be developed and tested.

## Appendix 1.

### Interview guide in the first wave of interviews

#### Questions for parents

What does your close family look like?

Please tell me about your family and what is going on in your family.

What is the general atmosphere in your family?

How is your illness affecting you and your family?

How is to be ill and parent? Please describe.

What does your family notice of your illness?

Do you speak about your illness in your family?

What has your child said about your illness?

What do you wish the clinic to do for you and your child?

What support do you wish? Please, give examples.

How can the clinic offer this support to you in the best way?

#### Questions for children

How is it to have a parent who is ill?

Please tell me about your family and what is going on in your family.

How do you feel when you think about your parent being ill?

Do you speak about your parent’s illness in your family?

What is the general atmosphere in your family?

How are you influenced by your parent’s illness?

What household chores do you do? Have they changed because of the illness?

Is there something you would like to ask your parent? Have you? If not, why?

What do you wish the clinic to do for you and your parent?

## Appendix 2.

### Interview guide in second wave of interviews

#### Questions for parents

What does your close family look like?

Has your child acted differently since you became ill?

Can you speak about your illness in the family?

What have you said about your illness to your child?

When do you speak?

How do you speak? Please give examples.

What is the greatest hindrance for speaking about the illness?

Do you have examples of when speaking about the illness has worked well?

What support would be helpful for you to be able to talk to your child about the illness?

When would you like the support?

From whom would you like the support?

#### Questions for children

What does your close family look like?

When did you notice that your parent was not feeling well?

How did you notice?

Do you know what illness your parent has?

How did you find out?

When do you talk about your parent’s illness in your family?

Can you ask your parent anything about the illness? Why not?

We would like to learn to help children who have an ill parent. Imagine you have a classmate with a sick parent. What would the classmate wish to know? When and how should the classmate get this information?
